# Passive Immunity Trial for Our Nation (PassITON): study protocol for a randomized placebo-control clinical trial evaluating COVID-19 convalescent plasma in hospitalized adults

**DOI:** 10.1186/s13063-021-05171-2

**Published:** 2021-03-20

**Authors:** Wesley H. Self, Thomas G. Stewart, Allison P. Wheeler, Wissam El Atrouni, Amanda J. Bistran-Hall, Jonathan D. Casey, Vince D. Cataldo, James D. Chappell, Claudia S. Cohn, Jessica B. Collins, Mark R. Denison, Marjolein de Wit, Sheri L. Dixon, Abhijit Duggal, Terri L. Edwards, Magali J. Fontaine, Adit A. Ginde, Michelle S. Harkins, Thelma Harrington, Estelle S. Harris, Daanish Hoda, Tina S. Ipe, Stuti J. Jaiswal, Nicholas J. Johnson, Alan E. Jones, Maryrose Laguio-Vila, Christopher J. Lindsell, Jason Mallada, Manoj J. Mammen, Ryan A. Metcalf, Elizabeth A. Middleton, Simon Mucha, Hollis R. O’Neal, Sonal R. Pannu, Jill M. Pulley, Xian Qiao, Jay S. Raval, Jillian P. Rhoads, Harry Schrager, Carl Shanholtz, Nathan I. Shapiro, Stephen J. Schrantz, Isaac Thomsen, Krista K. Vermillion, Gordon R. Bernard, Todd W. Rice, For the Passive Immunity Trial for Our Nation (PassITON) Investigators

**Affiliations:** 1 Vanderbilt Institute for Clinical and Translational Research (VICTR), Vanderbilt University Medical Center, 1313 21st Ave South, 312 Oxford House, Nashville, TN 37232 USA; 2 Department of Emergency Medicine, Vanderbilt University Medical Center, Nashville, USA; 3 Department of Biostatistics, Vanderbilt University Medical Center, Nashville, USA; 4 Department of Pathology, Microbiology, and Immunology, Vanderbilt University Medical Center, Nashville, USA; 5 Division of Infectious Diseases, Department of Internal Medicine, The University of Kansas School of Medicine, Kasas, USA; 6 Department of Medicine, Division of Allergy, Pulmonary, and Critical Care Medicine, Vanderbilt University Medical Center, Nashville, USA; 7 Division of Hematology and Oncology, Louisiana State University Health-Sciences Center, New Orleans, USA; 8 Division of Infectious Diseases, Department of Pediatrics, Vanderbilt University Medical Center, Nashville, USA; 9 Department of Laboratory Medicine and Pathology, University of Minnesota, Minneapolis, USA; 10 Division of Pulmonary Disease and Critical Care Medicine, Department of Internal Medicine, Virginia Commonwealth University, Richmond, USA; 11 Department of Critical Care, Respiratory Institute, Cleveland Clinical Healthcare System, Cleveland, USA; 12 Division of Transfusion Services, Department of Pathology, University of Maryland School of Medicine, Baltimore, USA; 13 Department of Emergency Medicine, University of Colorado School of Medicine, Boulder, USA; 14 Department of Medicine, University of New Mexico School of Medicine, Albuquerque, USA; 15 Division of Pulmonary and Critical Care Medicine, Department of Medicine, University of Maryland School of Medicine, College Park, USA; 16 Division of Pulmonary and Critical Care Medicine, Department of Medicine, University of Utah, Salt Lake City, USA; 17 Intermountain Healthcare, Salt Lake City, USA; 18 Department of Pathology and Laboratory Medicine, University of Arkansas for Medical Sciences, Fayetteville, USA; 19 Division of Hospital Medicine, Scripps Clinic, Scripps Research Translational Institute, The Scripps Research Institute, San Diego, USA; 20 Department of Emergency and Division of Pulmonary, Critical Care, and Sleep Medicine, University of Washington, Seattle, USA; 21 Department of Emergency Medicine, University of Mississippi Medical Center, Oxford, USA; 22 Department of Internal Medicine, Division of Infectious Disease, Rochester General Hospital, Rochester, USA; 23 Department of Pharmacy, Newton-Wellesley Hospital, Massachusetts College of Pharmacy and Health Sciences, Boston, USA; 24 Division of Pulmonary, Critical Care, and Sleep Medicine, Department of Medicine, State University of New York at Buffalo, Buffalo, USA; 25 Department of Pathology, University of Utah, Salt Lake City, USA; 26 Division of Pulmonary and Critical Care, Louisiana State University Health-Sciences Center, New Orleans, USA; 27 Division of Pulmonary, Critical Care, and Sleep Medicine, The Ohio State University, Columbus, USA; 28 Sentara Pulmonary, Critical Care, and Sleep Specialists, Sentara Health, Eastern Virginia Medical School, Norfolk, USA; 29 Department of Pathology, University of New Mexico School of Medicine, Albuquerque, USA; 30 Newton-Wellesley Hospital, Department of Medicine, Tufts School of Medicine, Boston, USA; 31 Department of Emergency Medicine, Beth Israel Deaconess Medical Center, Boston, USA; 32 Department of Medicine, University of Chicago, Chicago, USA

**Keywords:** COVID-19, SARS-CoV-2: convalescent plasma, Passive immunity, Neutralizing antibodies, Clinical trials, Randomized controlled trial

## Abstract

**Background:**

Convalescent plasma is being used widely as a treatment for coronavirus disease 2019 (COVID-19). However, the clinical efficacy of COVID-19 convalescent plasma is unclear.

**Methods:**

The *Pass*ive *I*mmunity *T*rial for *O*ur *N*ation (PassITON) is a multicenter, placebo-controlled, blinded, randomized clinical trial being conducted in the USA to provide high-quality evidence on the efficacy of COVID-19 convalescent plasma as a treatment for adults hospitalized with symptomatic disease. Adults hospitalized with COVID-19 with respiratory symptoms for less than 14 days are eligible. Enrolled patients are randomized in a 1:1 ratio to 1 unit (200–399 mL) of COVID-19 convalescent plasma that has demonstrated neutralizing function using a SARS-CoV-2 chimeric virus neutralization assay. Study treatments are administered in a blinded fashion and patients are followed for 28 days. The primary outcome is clinical status 14 days after study treatment as measured on a 7-category ordinal scale assessing mortality, respiratory support, and return to normal activities of daily living. Key secondary outcomes include mortality and oxygen-free days. The trial is projected to enroll 1000 patients and is designed to detect an odds ratio ≤ 0.73 for the primary outcome.

**Discussion:**

This trial will provide the most robust data available to date on the efficacy of COVID-19 convalescent plasma for the treatment of adults hospitalized with acute moderate to severe COVID-19. These data will be useful to guide the treatment of COVID-19 patients in the current pandemic and for informing decisions about whether developing a standardized infrastructure for collecting and disseminating convalescent plasma to prepare for future viral pandemics is indicated.

**Trial registration:**

ClinicalTrials.gov NCT04362176. Registered on 24 April 2020.

**Supplementary Information:**

The online version contains supplementary material available at 10.1186/s13063-021-05171-2.

## Background

Since emerging in late 2019, severe acute respiratory syndrome coronavirus 2 (SARS-CoV-2) has caused a global health crisis [1]. The disease caused by SARS-CoV-2 infection, coronavirus disease 19 (COVID-19), has caused over 2.1 million deaths worldwide through January 2021 [2]. Despite vast ongoing efforts to identify potential treatments for patients with acute COVID-19, few therapies have demonstrated benefit, and these drugs appear to only be effective for certain subgroups of patients with COVID-19 [3, 4]. The recent approval of two vaccines offers promise for preventing new infections in the future [5, 6]. However, logistics of manufacturing and deploying the vaccine worldwide appear challenging, especially in resource-poor and developing nations [7–9]. Additionally, many people appear reluctant to receive SARS-CoV-2 vaccines even when they do become widely available [10]. Furthermore, vaccines are unlikely to completely eliminate COVID-19 in vaccinated populations [11–14]. Thus, COVID-19 may be a major cause of morbidity and mortality for the foreseeable future and effective therapies to treat patients moderately and severely ill with COVID-19 are urgently needed.

### Rationale for convalescent plasma as a therapy for COVID-19

The use of convalescent plasma as a therapy for acute infections relies on the concept of transferring neutralizing antibodies from a person who recently recovered from the disease and developed a robust pathogen-specific immune response to another person who is in the early stages of the infection and has not fully developed his or her own immune response. This type of therapy is often called passive immune therapy or passive antibody therapy.

Based on strong biological rationale, convalescent plasma has been used for more than a century to treat outbreaks of viral diseases, especially when therapeutic options have been lacking [15, 16]. However, convalescent plasma therapy has rarely been evaluated by rigorously designed randomized trials, resulting in little empiric evidence to support its use. Argentine hemorrhagic fever is the only viral illness for which convalescent plasma was conclusively shown to be efficacious [17].

### Expanded access program and emergency use authorization

Despite limited data on efficacy, hundreds of thousands of patients have received COVID-19 convalescent plasma outside of a clinical trial. The expanded access program (EAP) in the US for COVID-19 convalescent plasma was started in April 2020. More than 90,000 patients were treated in through this program, which was primarily designed to provide access to convalescent plasma early in the pandemic and only secondarily to evaluate safety and effectiveness [18, 19]. Less than 1% of treated patients experienced a transfusion reaction [20]. Because the program did not include a control group, efficacy was assessed by comparing clinical outcomes among patients who received convalescent plasma with high, medium, and low levels of SARS-CoV-2 antibodies, using the concept that better clinical outcomes in patients who received plasma with higher antibody levels would suggest efficacy [21]. Antibody levels were retrospectively measured with the Ortho-Clinical Diagnostics VITROS IgG semiquantitative assay and classified into the following three groups: (1) high antibody level (signal-to-cutoff ratio > 18.45); (2) medium antibody level (signal-to-cutoff ratio 4.62–18.45); and (3) low antibody level (signal-to-cutoff < 4.62). Among 3082 patients who received a single unit of convalescent plasma with measured antibody levels, and thus could be assigned to a single category of antibody level, 30-day mortality varied in a “dose-dependent” pattern by antibody titer level: 22.3% mortality in the high titer group, 27.4% in the medium titer group, and 29.6% in the low titer group [19].

Citing results of the EAP, along with a small trial from China [22] and a trial from the Netherlands [23] that halted early, the Food and Drug Administration (FDA) issued an emergency use authorization (EUA) on August 23, 2020, for COVID-19 convalescent plasma to treat hospitalized COVID-19 patients [20]. At that time, the FDA concluded that existing evidence suggested that COVID-19 convalescent plasma with high antibody titer may be beneficial but emphasized that additional high-quality randomized clinical trials were important to more definitively understand the efficacy of COVID-19 convalescent plasma.

### Clinical trials of COVID-19 convalescent plasma

COVID-19 convalescent plasma trials published after the announcement of the EUA include the PLACID [24] and PlasmAR [25] trials conducted among hospitalized adults in India and Argentina, respectively, and another trial of older outpatient adults in Argentina [26].

Neither PLACID nor PlasmAR suggested efficacy for COVID-19 convalescent plasma. PLACID enrolled 464 patients randomized to convalescent plasma administered in two 200 mL doses versus usual care in an unblinded fashion. Neutralizing capacity of the plasma was measured retrospectively and less than one-third of the units transfused in the study possessed neutralizing antibody titers ≥ 1:80 by a microneutralization assay. The randomized, blinded, placebo-controlled PlasmAR trial enrolled 335 patients in a 2:1 convalescent plasma-to-placebo ratio. Convalescent plasma units were chosen for transfusion if they were found to have SARS-CoV-2 IgG titers 1:800 by the COVIDAR assay. Retrospective analysis of neutralizing titers in 125 (56%) of the infused doses showed an 80% inhibitory concentration median titer of 1:300. Treatment with convalescent plasma in this trial did not significantly improve clinical status at 30 days as measured on a six-level ordinal scale. A subsequent meta-analysis of available observational studies and clinical trials involving hospitalized patients suggested potential efficacy for COVID-19 convalescent plasma [27].

Libster et al. conducted a randomized trial of 160 outpatients who were ≥ 65 years old with mild COVID-19 and symptoms < 72 h [26]. Participants were randomized in a 1:1 ratio to “high titer” COVID-19 convalescent plasma (IgG titer greater than 1:1000 against the spike protein) or placebo. Progression to severe respiratory disease, defined as a respiratory rate ≥ 30 breaths/min or oxygen saturation < 93% while breathing room air, occurred in fewer patients randomized to convalescent plasma (16%) than placebo (31%), suggesting COVID-19 convalescent plasma treatment may be efficacious for early, mild disease [26].

Results from these trials published after the FDA emergency use authorization were unknown at the time that PassITON was designed. However, it is noteworthy that unlike PassITON, many prior studies of COVID-19 convalescent plasma either did not quantify SARS-CoV-2 antibody titers or screened convalescent plasma units for SARS-CoV-2 antibodies with binding assays without testing for neutralization. Among patients with high detectable levels of SARS-CoV-2 antibodies, only approximately 40–50% appear to have neutralizing function [28]. Therefore, many prior COVID-19 convalescent plasma studies likely included plasma units without neutralizing function.

### Goal of this trial

Rigorous clinical trials evaluating the efficacy COVID-19 convalescent plasma with neutralizing activity are needed to guide clinical practice regarding the use of convalescent plasma during the current pandemic and also to understand if developing a scalable infrastructure for collecting, testing, and disseminating convalescent plasma is an important investment to prepare for future outbreaks of novel viruses. Convalescent plasma could be an immediately available therapy in the early stages of future viral pandemics in resource-rich and resource-limited nations. Understanding the efficacy of convalescent plasma in the current COVID-19 pandemic could help inform decisions on pursuing convalescent plasma as a therapy for future pandemics.

This study—the *Pass*ive *I*mmunity *T*rial for *O*ur *N*ation (PassITON)—was designed to provide the highest quality evidence on the efficacy of COVID-19 convalescent plasma as a therapy for adults hospitalized with moderate-to-severe acute COVID-19.

## Methods

### Design and oversight

PassITON is a multicenter, blinded, placebo-controlled, parallel group, randomized clinical trial evaluating the efficacy of COVID-19 convalescent plasma with neutralizing antibodies for the treatment of adults hospitalized with acute COVID-19. The trial is funded by the National Center for Advancing Translational Sciences (NCATS—https://ncats.nih.gov/contact) of the National Institutes of Health (NIH). NCATS played no part in study design, collection, management, analysis, or interpretation of data, writing of the report, or the decision to submit the report for publication. The trial is conducted at hospitals across the USA including both academic medical centers (many of which are part of the NCATS Clinical and Translational Science Awards (CTSA) Program) and regional/city hospitals. All US hospitals with sufficient research enabling infrastructure are eligible to be a site in PassITON regardless of size or location.

Vanderbilt University Medical Center serves as the clinical coordinating center, data coordinating center, and single institutional review board (IRB number: 201672). The PassITON study team is located at Vanderbilt University Medical Center and is responsible for direct trial oversight via twice weekly meetings. This team is comprised of the study principal investigators, statisticians, project managers, operations managers, content experts, and study coordinators. Protocol-related changes, inclusion of ancillary studies, and other high-level decision-making are conducted by the PassITON Steering Committee (all site principal investigators) at biweekly meetings.

Treatment with convalescent plasma in this trial is through an investigational new drug (IND number 21080) application submitted to the FDA. The trial was registered with ClinicalTrials.gov (NCT04362176) prior to enrollment of the first participant on April 28, 2020. Progress and safety of the trial is monitored by an independent Data and Safety Monitoring Board (DSMB).

Prior to initiation of study procedures, informed consent is obtained by a trained study coordinator or investigator from each patient or a legally authorized surrogate decision-maker if the patient is unable to make medical decisions. On the consent form, participants are asked if they agree to use of their data should they choose to withdraw from the trial. Participants are also asked for permission for the research team to share relevant data with people from the sites taking part in the research or from regulatory authorities, where relevant. This trial does not involve collecting biological specimens for storage. Consent is obtained electronically or on paper. There is no anticipated harm from participating in this trial or provision for harm-related compensation.

The trial protocol was developed according to the SPIRIT guidelines (Supplementary Materials, Supplemental Figure 1). Protocol modifications and changes to study-related procedures are communicated to the study team and investigators through twice weekly internal coordinating center team meetings, weekly PassITON newsletters disseminated both internally and externally to site staff and investigators, and biweekly Steering Committee meetings attended by site investigators and coordinators.

### Collection of convalescent plasma

**Fig. 1:**
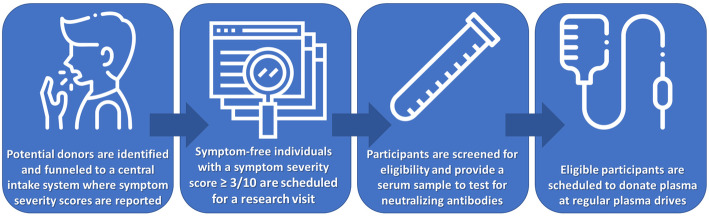
Donor selection pipeline for obtaining COVID-19 convalescent plasma for PassITON. Images obtained from flaticon.com

The plasma collection component of PassITON was developed to optimize the efficient procurement of COVID-19 convalescent plasma with high levels of neutralizing antibodies. Convalescent plasma is collected from adults mainly residing around Nashville, Tennessee who have recovered from COVID-19 in a collaborative effort between Vanderbilt University Medical Center and Blood Assurance, a nonprofit regional blood center based in Chattanooga, Tennessee. Patients with laboratory-confirmed SARS-CoV-2 infection with self-reported symptom severity of at least 3 on a 10-point scale (range: 1, “I feel healthy” to 10, “I was/should have been in the Intensive Care Unit (ICU)”) are eligible for plasma donation. Recovered patients are identified through several methods including Vanderbilt hospital records, mass email through the Vanderbilt employee list, public advertising in the community, Research-Match [29, 30], and self-identification. Patients are able to donate plasma if they have recovered from acute COVID-19, defined as either: (1) being symptom-free for 14 days and having at least one negative COVID-19 test by RT-PCR, or (2) being symptom-free for at least 28 days. All donors must also meet FDA requirements for blood product donation [31]. Donors sign an IRB-approved informed consent for participation prior to phlebotomy. Donors have a blood sample collected for characterization of circulating SARS-CoV-2 antibodies (see next section) and then immediately have blood collected for plasma donation units (Fig. 1).

Plasma collection is performed via apheresis using the Fresenius-Kabi ALYX instrument, which allows for the collection of up to four units per donation. Patients are invited to return for additional donations if antibody testing demonstrates high antibody levels (≥ 20,000 EU/mL by anti-Receptor Binding Domain (RBD) IgG binding assay) and neutralizing activity. Through an FDA variance obtained by Blood Assurance, participants are allowed to donate as frequently as every 7 days for 4 visits before evaluation of total protein and serum albumin to confirm safety of continued donations.

### Selection of convalescent plasma for the trial

Serum is obtained from convalescent plasma donors at the time of donation. These serum samples are used for antibody quantification. Donor sera are initially screened by the Abbott™ ARCHITECT™ IgG qualitative platform for the presence of detectable antibody against SARS-CoV-2. Samples positive in the Abbott assay are then assessed by the quantification of binding IgG against the RBD of SARS-CoV-2 using a liquid bead-array assay as previously described [32]. Briefly, recombinant RBD is conjugated to Luminex MagPlex microspheres and incubated in 96 well plates with serially diluted serum samples and a cross-reactive SARS-CoV-2 monoclonal antibody as a standard. Serum antibodies bound to SARS-CoV-2 S_RBD_ are detected by R-Phycoerythrin conjugated F(ab’)2 fragment goat anti-human IgG Fc gamma conjugate (Jackson ImmunoResearch). Data are acquired using a Luminex MagPix Instrument at 100 beads per well, with Xponent software version 4.3.

**Fig. 2:**
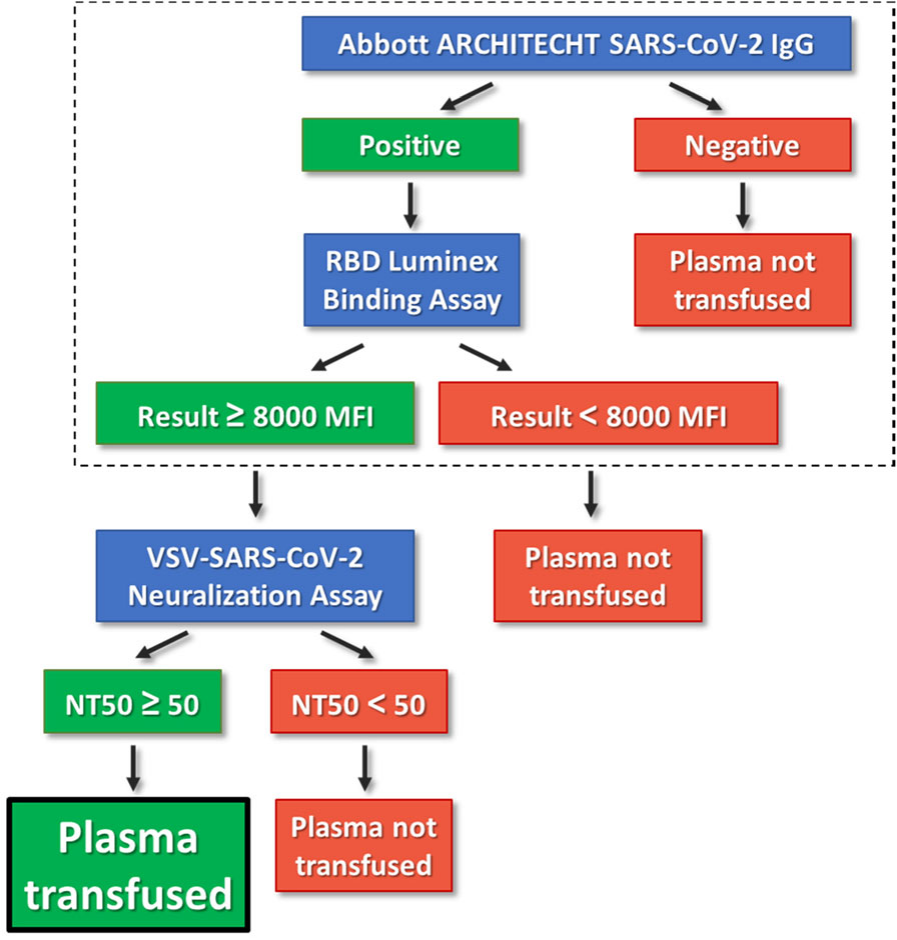
COVID-19 convalescent plasma selection for PassITON. Each convalescent plasma unit used in the trial is selected based on quantitative anti-SARS-CoV-2 antibody levels and neutralization

Through September 30, 2020, donor samples were screened exclusively using the Abbott™ ARCHITECT™ platform and the RBD Luminex assay (Fig. 2). Units with a minimum threshold mean fluorescence intensity (MFI) of 8000 were deemed eligible for transfusion. This cutoff was determined by screening a subset of samples for neutralization using a traditional live-virus plaque-reduction neutralization titer (PRNT) assay. Two- or four-fold serial dilutions of sera in gelatin saline were incubated for 20 min at 37 °C with an average of 130 plaque-forming units of SARS-CoV-2 isolate SARS-CoV-2/human/USA/USA-WA1/2020 (GenBank: MN985325.1) in 200 μl gelatin saline, and 100 μl of virus-serum mixtures were applied to each of two Vero E6 cell monolayers in 10 cm^2^ dishes. Following virus adsorption for 30 min at 37 °C, monolayers were overlaid with 1% agar in cell culture medium and incubated for 3 days at 37 °C, at which time plaques were enumerated by direct visual inspection. Percent neutralization was defined as fractional plaque reduction in the presence of serum relative to untreated (saline only) virus. Neutralization titers were interpolated from dose-response curves fit to results of duplicate neutralization testing using five-parameter logistic regression modeling implemented in GraphPad Prism.

Beginning October 1, 2020, an additional screening step was introduced to confirm the presence of SARS-CoV-2 neutralizing antibodies. In the modified format, donor samples still undergo screening via the Abbott™ ARCHITECT™ platform and the RBD Luminex assay. Samples are excluded if they are found to be negative via the Abbott™ ARCHITECT™ of having an MFI < 8000 as determined by the RBD Luminex assay. Samples with an antibody level (MFI) above 8000 are then screened for the ability to neutralize virus by functional assessment with a high-throughput assay platform using real-time, quantitative cellular analysis on the xCELLigence platform (Agilent Technologies, Santa Clara, CA), using chimeric vesicular stomatitis virus (VSV) expressing intact SARS-CoV-2 spike protein, as previously described [33, 34]. Samples with a 50% neutralization titer > 1:50 are selected for transfusion in the trial (Fig. 2).

Antibody testing is completed at Vanderbilt University Medical Center. Convalescent plasma units are stored at Blood Assurance and shipped to enrolling sites in the trial as needed. Between April 22, 2020, and January 29, 2021, 429 donors provided over 1200 convalescent plasma units, with plans to continue donations for the duration of the trial. Approximately 25% of the plasma donations in this program have passed the antibody screening steps and been selected for use in the trial.

### Trial participants

Patients eligible for enrollment in the trial include adults hospitalized with laboratory-confirmed SARS-CoV-2 infection and respiratory symptoms consistent with COVID-19 for fewer than 14 days. Patients hospitalized in either ICU or less intensive areas are eligible. Major exclusion criteria include planned hospital discharge within 24 h and prior receipt of COVID-19 convalescent plasma or another passive immunity therapy in the prior 30 days. Participants identified as eligible for the study are approached for consent by a study coordinator or investigator. Recruitment materials include a flyer, fact sheet, and website (https://passitonstudy.org) which contains informative videos from physicians, researchers, and past participants. All recruitment materials have been translated to Spanish, Arabic, and simple Chinese. To further support recruitment, there are fully translated consent forms in > 20 languages. PassITON recruitment will continue at all sites until the accrual goal of 1000 participants is met.

### Randomization and treatment groups

Enrolled patients are randomized in a 1:1 ratio to COVID-19 convalescent plasma or placebo. Randomization is completed by a centralized web-based platform and stratified by site, sex, and age. Patients randomized to convalescent plasma receive a single dose of 1 unit (200–399 mL) of COVID-19 convalescent plasma infused intravenously. Patients randomized to placebo receive a single 250 mL dose of lactated Ringer’s solution containing multivitamin infused intravenously. Multivitamins are added to the placebo solution to produce a yellow color that matches the color of plasma.

The study infusion (convalescent plasma or placebo) is administered as soon as possible and within 24 h after randomization. Infusion of study treatment is halted if the study participant exhibits any symptoms of transfusion reaction or anaphylaxis. Patients are observed for 6 h after initiation of the study infusion for signs and symptoms of a transfusion reaction. Use of open-label convalescent plasma is strongly discouraged for the first 14 days following the study infusion. Other aspects of clinical management are performed at the discretion of the treating clinicians without influence from the study protocol.

### Blinding

In order to safely administer a blood product in the trial and also maintain blinding of the patient, investigators, and outcome assessors, the trial uses both blinded and unblinded study personnel. At each site, the lead investigator remains blinded to study group assignment. An unblinded study member randomizes patients, receives the treatment assignment, and then orders convalescent plasma or the placebo solution based on the randomized treatment assignment. The study treatment is delivered to the patient’s bedside, where an unblinded clinical nurse places the study treatment in a blinding bag before entering the patient’s room. The unblinded clinical nurse then infuses the treatment. Clinical monitoring, including vital sign assessment, is completed based on local practices for monitoring an infusion of plasma regardless of randomized group. The clinical providers (e.g., physicians), patient, and outcome assessors remain blinded to study group assignment. Participant unblinding is performed by unblinded site coordinators and is permitted only after the study follow-up period is complete and if unblinding will directly impact the individual’s course of clinical care (e.g., timing of COVID-19 vaccination).

### Outcomes

The primary outcome is the patient’s clinical status on a 7-category ordinal scale (the COVID-19 7-point Ordinal Clinical Progression Outcomes Scale) 14 days after the study infusion. The 7 categories are (1) not hospitalized with resumption of normal activities; (2) not hospitalized, but unable to resume normal activities; (3) hospitalized, not on supplemental oxygen; (4) hospitalized, and on supplemental oxygen; (5) hospitalized, on nasal high-flow oxygen therapy, noninvasive mechanical ventilation, or both; (6) hospitalized, on ECMO, invasive mechanical ventilation, or both; and (7) death. While the patient is hospitalized, the ordinal scale category is identified by direct patient observation and medical record review. After hospital discharge, patients are contacted by telephone to distinguish between category 1 and category 2. This scale was developed by the World Health Organization [35] (WHO) early in the pandemic as a patient-centered clinical outcome for COVID-19 and has been successfully used in multiple clinical trials [3, 36, 37]. Secondary and safety outcomes are shown in Table 1.

**Table 1:** Trial outcomes. Definitions for all outcome are provided in the statistical analysis plan available in the Supplementary Materials

Outcomes	Variable type	Approach to analysis
Efficacy outcomes		
COVID-19 7-point Ordinal Clinical Progression Outcomes Scale 14 days after randomization (assessed on study day 15)	Ordinal	Cumulative probability model with logit link
All-location, all-cause 14-day mortality (assessed on study day 15)	Binary	Logistic regression
All-location, all-cause 28-day mortality (assessed on study day 29)	Binary	Logistic regression
Survival through 28 days	Time-to-event	Proportional hazards regression
Time to hospital discharge through 28 days	Time-to-event	Multistate model with death as a competing risk
Time to recovery	Time-to-event	Cumulative probability model with logit link
COVID-19 7-point Ordinal Clinical Progression Outcomes Scale on study days 3, 8 and 29	Ordinal	Cumulative probability model with logit link
Oxygen-free days through day 28	Ordinal	Cumulative probability model with logit link
Ventilator-free days through day 28	Ordinal	Cumulative probability model with logit link
Vasopressor-free days through day 28	Ordinal	Cumulative probability model with logit link
ICU-free days through day 28	Ordinal	Cumulative probability model with logit link
Hospital-free days through day 28	Ordinal	Cumulative probability model with logit link
Safety outcomes		
Receipt of renal replacement therapy	Binary	Risk difference
Venous thromboembolic disease (deep vein thrombosis or pulmonary embolism)	Binary	Risk difference
Cardiovascular event (myocardial infarction or ischemic stroke)	Binary	Risk difference
Transfusion reaction	Binary	Risk difference
Transfusion related acute lung injury (TRALI)	Binary	Risk difference
Transfusion associated circulatory overload (TACO)	Binary	Risk difference
Transfusion-related infection	Binary	Risk difference

### Data collection, monitoring, and dissemination

Randomization and data collection are being conducted through Research Electronic Data Capture (REDCap). The randomization module in REDCap allows the statistician to load a randomization table that will allow the study personnel to click a “randomize” button. REDCap is a secure, web-based application designed to support data capture for research studies, providing (1) an intuitive interface for validated data entry; (2) audit trails for tracking data manipulation and export procedures; (3) automated export procedures for seamless data downloads to common statistical packages; and (4) procedures for importing data from external sources.

Data quality is reviewed remotely using front-end range and logic checks at the time of data entry and back-end monitoring of data using application programming interface tools connecting the online database to statistical software to generate data reports. Patient records and case report forms are also be reviewed to evaluate the accuracy and completeness of the data entered into the database and monitored for protocol compliance per the study monitoring plan.

The data generated from the PassITON trial will be released via publication. It will also be shared at seminars, symposiums, and meeting presentations as well as deposited in appropriate databases. Before releasing any of this information, the raw data will be stripped of identifiers in order to remain compliant with HIPAA and other governing agencies’ guidelines. The datasets analyzed during the current study are available from the corresponding author upon reasonable request.

### Statistical analysis

In this section, we describe key statistical features of the trial. The full statistical analysis plan for the trial is included in the Supplemental Materials.

#### General approach to analysis

The statistical design for this trial was informed by the need to learn as rapidly as possible from the data during the pandemic while simultaneously managing the risk of drawing erroneous conclusions. Rapid decision-making to maximally inform clinical care during an ongoing pandemic requires flexibility for the DSMB to perform unplanned evaluations of the data and potentially decrease or increase the sample size of the trial. This requires a trial framework that does not demand that all possible interim analyses are prespecified, as is required of approaches using *p* values. Two closely related approaches which offer the needed flexibility are the Likelihood and Bayesian frameworks. We selected the Likelihood framework for this trial. The Likelihood approach has been successfully implemented in clinical trials with continuous monitoring or sequential methods [38, 39], because it retains its meaning and reliability regardless of the number of interim analyses or outcomes under consideration [40, 41].

Decision-making using the likelihood approach in a clinical trial center on three quantities: the point estimate of the treatment effect (an odds ratio, for example), a corresponding interval estimate, and a single number summary that measures the relative evidence for one hypothesis (for example, convalescent plasma being superior to placebo) compared to another hypothesis (for example, convalescent plasma not being superior to placebo). These three quantities are similar to the point estimate, 95% confidence interval, and *p* value that are generated in frequentist analyses. In fact, point estimates using the likelihood and frequentist approaches are often identical, and the interval estimates are often very similar to 95% confidence intervals. The likelihood ratio (LR) and the *p* value, however, are distinct measures of evidence. The LR is a ratio: the density of the trial data if the treatment is effective (alternative hypothesis) divided by the density of the trial data if the treatment is not effective (null hypothesis). A LR of 1 indicates the data are neutral; neither the alternative hypothesis nor null hypothesis is supported more strongly than the other. A large LR is evidence in support of the treatment being effective. An LR less than one is evidence that the treatment is harmful. In this trial, a LR ≥ 7 in favor of the intervention group is considered sufficient evidence to assert that the treatment is beneficial.

The likelihood approach is different than using *p*-values as the level of evidence because the p-value compares what actually happened in the trial to what might have happened if the trial were repeated infinitely and the null hypothesis was true. Because it is impossible to compute what might have happened if the rules for decision-making are not fully predefined, using a *p*-value for decision making is not well suited for a trial like this in which pandemic circumstances prompt urgent design changes. The LR approach, on the other hand, is based on a relative likelihood of observed outcomes under two competing models at the same point in time, making it especially appropriate for settings where pre-specification of the timing or frequency of sequential analyses is not possible.

#### Interim analyses

The anticipated sample size is 1000 enrolled patients. The trial includes three planned interim analyses, to be conducted after primary outcome data collection is completed for 150, 450, and 750 study participants. Additional interim analyses may be called at any time by the DSMB based on changes in the pandemic and/or emerging data on COVID-19 convalescent plasma. Adverse events, safety outcomes, protocol deviations, and the primary endpoint are presented to the DSMB at each interim analysis. Additionally, as a safety evaluation, the difference in mortality risk between groups is calculated, and the one-sided hypothesis that mortality risk in the intervention arm exceeds the mortality risk in placebo will be compared to the null hypothesis of equal mortality risk. The trial will be stopped for safety if the likelihood ratio for mortality exceeds any of the following thresholds, suggesting increased mortality with convalescent plasma: first interim analysis, LR 6.3 (which corresponds to a *p* value of approximately 0.0275); second interim analysis, LR 4.0 (which corresponds to a *p* value of approximately 0.0479); and third interim analysis, LR 3.3 (which corresponds to a *p* value of approximately 0.0612). These thresholds result in a 0.1 trial-wise risk of stopping the trial early for mortality if mortality were truly equivalent in the intervention and control groups. There are no pre-specified stopping rules for efficacy.

#### Primary analysis of the primary outcome

The primary analysis will be intention-to-treat, with each randomized patient analyzed according to the randomized treatment assignment (convalescent plasma vs. placebo) regardless of the treatment received. The main result will be an estimate of the treatment effect odds ratio, its likelihood ratio when compared to the null, and the corresponding 1/7 likelihood support interval, all of which will be estimated from a cumulative probability ordinal regression model (CPM) with logit link. The marginal likelihood function for the treatment effect parameter will be the asymptotic regression coefficient distribution; specifically, it will be the normal distribution density function with mean and standard deviation equal to the regression estimates. An odds ratio < 1.0 indicates more favorable results on the COVID 7-point Ordinal Clinical Progression Outcomes Scale in the intervention group compared with the control group. Likelihood ratios more extreme than 7 will be interpreted as sufficient evidence to assert efficacy.

The primary model will adjust for the following six baseline characteristics: age (2 parameters, restricted cubic spline); sex (1 parameter); baseline SOFA score (1 parameter, linear term); baseline COVID-19 7-point Ordinal Clinical Progression Outcomes Scale score (possible range: 3–6) (2 parameters, quadratic); time from symptom onset to randomization in days (2 parameter, non-linear term); and a site indicator variable (as a random effect).

#### Additional analyses of the primary outcome

A per-protocol analysis of the primary outcome will be performed in which randomized patients who did not receive any volume of the study treatment are excluded.

The impact of convalescent plasma quality, as measured by antibody quantification and neutralization, on the primary outcome will be estimated with two ordinal regression models. In the first, the model will include the same covariates listed for the primary analysis with the addition of a measure of donor plasma binding level (value in MFI obtained using the RBD Luminex-based assay [32]). In the second model, a measure of donor plasma neutralization (NT50 value obtained using the VSV-SARS-CoV-2 chimeric virus neutralization assay [33, 34]) will be used. Both of these variables of convalescent plasma quality will be included in the models as a restricted cubic spline with three knots to capture potential non-linear associations with the outcome. For observations in the control arm, binding and neutralization values will be set to zero. Studies to evaluate alternative measures of convalescent plasma quality are ongoing and, dependent on the results of those analyses, a different measure of quality may be selected.

The degree to which pre-specified baseline variables modify the treatment effect will be examined with tests of statistical interaction in a cumulative probability ordinal regression model. Independent variables will include study group assignment, the potential effect modifier of interest, the interaction between the two, and the same pre-specified covariates used in the primary model. Presence of effect modification will be assessed by reference to the LR for the interaction term, with values greater than 6 considered to suggest a potential interaction and values greater than 7 considered to confirm an interaction. The baseline variables that will be evaluated for effect modification include: baseline recipient (trial participant) serum antibody quantification; baseline COVID-19 7-point Ordinal Clinical Progression Outcomes Scale score; baseline SOFA score; location at time of enrollment (ICU/ward); age; race/ethnicity; duration of COVID-19 symptoms prior to randomization (days in linear form); and mechanical ventilation status at baseline.

#### Sample size and power

The operating characteristics of the trial design were estimated by simulating study data to reflect different treatment effect sizes. Simulations for study power were performed on Amazon Elastic Compute Cloud servers using R version 4.0.2 [42] with the following packages: ordinal [43], magrittr [44], and doRedis [45]. The simulated study dataset was evaluated according to the stopping rule and analysis plan described above. For each effect size, 1000 simulated datasets were analyzed. A type I error occurred if the study asserted efficacy when in fact there was no treatment effect. The type I error rate was calculated as the proportion of simulated studies with no treatment effect in which the error occurred. A type II error occurred if the study failed to assert efficacy when there was a beneficial treatment effect. For each treatment effect, power was calculated as the proportion of simulated studies that did not result in a type II error.

The study endpoint for control subjects was simulated to match the outcomes in the control arm of a recent clinical trial [46]. In each simulation setting, the distribution for the treatment arm was calculated by adjusting the control arm outcome distribution according to the setting-specific treatment effect size and data generation model.

**Fig. 3:**
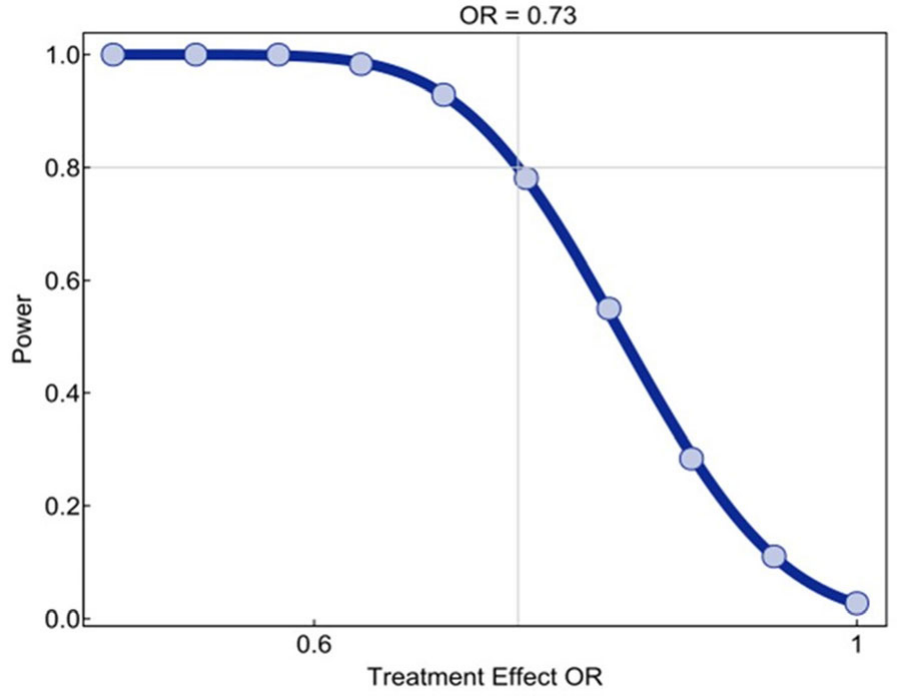
Power of the trial to detect different effect sizes (treatment effect odds ratios) in the primary outcome with enrollment of 1000 participants. The primary outcome is the COVID-19 7-point Ordinal Clinical Progression Scale, analyzed with a cumulative probability model. An odds ratio < 1.0 indicates more favorable clinical outcomes on the scale in the convalescent plasma group than the placebo group. With enrollment of 1000 participants, the trial has power to detect on odds ratio ≤ 0.73

These simulations demonstrated that enrollment of 1000 patients (500 patients in the intervention group and 500 patients in the control group) would provide 80% power to detect an adjusted odds ratio of ≤ 0.73 (Fig. 3). Some trials orient the ordinal outcomes scale in the reverse direction, with an odds ratio greater than 1.0 indicating benefit from the intervention [3, 36]. With reversal of the ordinal outcomes scale, enrollment of 1000 patients would provide 80% power to detect an adjusted odds ratio ≥ 1.37. The simulations also demonstrated that the type I error rate was below 0.05.

#### Analysis of secondary and safety outcomes

Secondary efficacy outcomes will be assessed by intention-to-treat analyses using the same covariables as the primary model for the primary outcome. Safety outcomes will be analyzed in the safety population, classified based on receipt of convalescent plasma from the trial vs. those who received placebo from the trial regardless of randomized assignment, without covariable adjustment. Adjustments will not be made for multiple comparisons.

## Discussion

PassITON is an ongoing blinded, placebo-controlled randomized trial evaluating the efficacy of COVID-19 convalescent plasma for the treatment of adults hospitalized with moderate-to-severe acute COVID-19. The first patient was enrolled on April 28, 2020, and trial completion is anticipated in 2021.

COVID-19 convalescent plasma has been administered to hundreds of thousands of patients in the USA, initially under an EAP [47] and now under EUA [20]. While data from these programs support the safety of convalescent plasma, evidence of efficacy is lacking. PassITON is designed to provide rigorous efficacy data. As such, key design features include meticulous selection of convalescent plasma with high levels of anti-SARS-CoV-2 antibodies with neutralizing activity; enrollment of a geographically diverse patient population in hospitals across the USA; patient-level randomization to COVID-19 convalescent plasma or matching placebo; blinding of participants, investigators and outcome assessors to treatment assignment; and systematic collection of patient-centered outcomes for 4 weeks following infusion of the study treatment. These characteristics distinguish PassITON from many other COVID-19 convalescent plasma trials which have either reported null findings (PLACID [24], PlasmAR [25]) or recently stopped for futility [48, 49].

In addition, the rigorous plasma donation and screening program required in PassITON to ensure the use of COVID-19 convalescent plasma with potent neutralizing antibodies has demonstrated important challenges that would need to be overcome to develop a scalable pipeline for supplying neutralizing COVID-19 convalescent plasma if it is found to be an effective therapy. With only approximately 25% of units donated from patients who have recovered from COVID-19 having neutralizing activity, stringent donor screening and antibody quantification steps would need to be implemented to ensure the use of effective convalescent plasma.

PassITON evaluates the efficacy of COVID-19 convalescent plasma among hospitalized patients, which is the same population described in the FDA EUA [20]. The efficacy of COVID-19 convalescent plasma among patients with less severe disease and/or who are earlier in their disease course is being evaluated in other trials [50, 51].

Convalescent plasma had been used as a therapy for severe viral illnesses for over a century and in hundreds of thousands of COVID-19 patients during the past year. Despite this enthusiastic use of convalescent plasma, its clinical efficacy remains unclear. Effectively managing COVID-19 patients in the current pandemic and developing a robust infrastructure to respond to future viral pandemics requires evidence-based answers to long-standing questions about the efficacy of convalescent plasma. PassITON will advance our understanding of convalescent plasma, and combined with other work, help inform treatment options for COVID-19 and future pandemics.

## Trial status

The PassITON trial launched in April 2020 as a single-center study at Vanderbilt University Medical Center with funding from the Dolly Parton COVID-19 Research Fund. The trial expanded to a multicenter study in August 2020 with funding from the NCATS of the National Institutes of Health (NIH). Prior to expansion to the multicenter format, 66 participants had been enrolled at Vanderbilt. Interim analyses conducted by the independent DSMB after enrollment and completion of the primary outcome by 151 and 469 study participants resulted in recommendations to continue the study without modification. PassITON is currently enrolling under protocol version 5.0 dated September 15, 2020. Trial completion is expected by May 31, 2021.

## Supplementary Information

**Additional file 1.** SPIRIT Checklist.

**Additional file 2.** Statistical Analysis Plan.

**Additional file 3: Supplemental Figure 1.** SPIRIT figure.

## Data Availability

Not applicable.

## Abbreviations

SARS-CoV-2: Severe acute respiratory syndrome coronavirus 2
COVID-19: Coronavirus disease 19
CTSA: Clinical and Translational Science Award
DSMB: Data Safety Monitoring Board
EAP: Expanded access program
EUA: Emergency use authorization
FDA: Food and Drug Administration
ICU: Intensive care unit
IND: Investigational new drug
IRB: Institutional Review Board
LR: Likelihood ratio
NCATS: National Center for Advancing Translational Science
NIH: National Institutes of Health
PassITON: Passive Immunity Trial for Our Nation
PRNT: Plaque Reduction Neutralization Titer
RBD: Receptor Binding Domain
VSV: Vesicular somatitis virus
